# Prevalence of and factors associated with post-traumatic stress disorder among French university students 1 month after the COVID-19 lockdown

**DOI:** 10.1038/s41398-021-01438-z

**Published:** 2021-05-27

**Authors:** Marielle Wathelet, Thomas Fovet, Améliane Jousset, Stéphane Duhem, Enguerrand Habran, Mathilde Horn, Christophe Debien, Charles-Edouard Notredame, Thierry Baubet, Guillaume Vaiva, Fabien D’Hondt

**Affiliations:** 1 Fédération de Recherche en Psychiatrie et Santé Mentale des Hauts-de-France, F-59000 Lille, France; 2 Centre National de Ressources et de Résilience Lille-Paris (CN2R), F-59000 Lille, France; 3 Department of Psychiatry, CHU Lille, F-59000 Lille, France; 4 Univ. Lille, Inserm, CHU Lille, U1172 - LilNCog - Lille Neuroscience & Cognition, F-59000 Lille, France; 5 Department of Public Health, CHU Lille, F-59000 Lille, France; 6 Univ. Lille, Inserm, CHU Lille, CIC1403 - Clinical Investigation Center, Lille, France; 7 Fonds FHF Recherche et Innovation, F-75993 Paris, France; 8 AP-HP, Avicenne Hospital, Department of Infant, Child and Adolescent Psychiatry, Sorbonne Paris Nord University, CESP, Bobigny, F-93000 France

**Keywords:** Scientific community, Psychiatric disorders

## Abstract

The COVID-19 pandemic and quarantine measures have sparked debate regarding their traumatic nature. This cross-sectional study reports the prevalence rate of probable post-traumatic stress syndrome (PTSD) and associated factors among French university students. A total of 22,883 students completed the online questionnaire. The prevalence rate of probable PTSD, assessed using the PTSD Checklist for DSM-5, was 19.5% [19.0–20.0]. Female (1.32 [1.21–1.45]) or non-binary gender (1.76 [1.35–2.31]), exposure to a non-COVID-19-related traumatic event (3.37 [3.08–3.67]), having lived through quarantine alone (1.22 [1.09–1.37]), poor quality of social ties (2.38 [2.15–2.62]), loss of income (1.20 [1.09–1.31]), poor quality housing (1.90 [1.59–2.26]), low-quality of the information received (1.50 [1.35–1.66]) and a high level of exposure to COVID-19 (from 1.38 [1.24–1.54] to 10.82 [2.33–76.57] depending on the score) were associated with PTSD. Quarantine was considered potentially traumatic by 78.8% of the students with probable PTSD. These findings suggest the pandemic context and lockdown measures could have post-traumatic consequences, stimulating debate on the nosography of PTSD.

## Introduction

The COVID-19 pandemic has led many countries to impose lockdown measures on their populations. As of April 2, 2020, 3.9 billion people were confined. In France, the first lockdown measure prohibited any movement deemed non-essential from March 17 to May 11, 2020. This unprecedented situation raised many concerns regarding the potential consequences of the pandemic context and lockdown measures on mental health. Indeed, previous studies have reported many negative psychological effects of quarantine, including post-traumatic stress symptoms^1^.

In the fifth edition of the American Psychiatric Association’s *Diagnostic and Statistical Manual of Mental Disorders* (DSM-5), post-traumatic stress disorder (PTSD) is said to belong to the category of trauma- and stressor-related stress disorders^2^. The core features of PTSD include four distinct diagnostic clusters: re-experiencing, avoidance, negative cognition/mood, and hyperarousal^2^. While there is growing evidence that PTSD may be an outcome of COVID-19-related events (e.g., the hospitalization or death of a relative), the occurrence of PTSD in the general population as a result of the pandemic and lockdown context is still a matter of debate^3^.

During the initial stage of the COVID-19 pandemic, several studies found high rates of moderate or severe distress symptoms in university students, a high-risk population for mental health conditions^4,5^: 53.8% in the Chinese study of Wang et al. and 28.1% in the French study of Wathelet et al.^6,7^. Importantly, while evidence suggests that many people experiencing acute stress reactions after a traumatic event will not develop PTSD^8^, no study analyzed response trajectories to the pandemic context. Furthermore, the first studies assessing the prevalence of PTSD among home-quarantined university students yielded very heterogeneous results, from 2.7^9^ to 30.8%^10^. Of note, these studies used either the full or the abbreviated version of the PTSD Checklist *–* Civilian Version (PCL-C), which has already yielded widely varying prevalence estimates in similar samples^11^. The PCL-C is based on the DSM-IV criteria for PTSD and hence does not fully overlap with the new 20-item PCL mapped to the DSM-5 (PCL-5), which exhibits a good test–retest reliability among trauma-exposed college students^12,13^.

In this study, we investigated the prevalence of and factors associated with probable PTSD among French university students 1 month after the first COVID-19 lockdown. We also analyzed which of the COVID-19-related events were subjectively considered as traumatic by university students and assessed the different psychological response trajectories following the first lockdown measure.

## Methods

### Population and study design

This study used the data of the repeated cross-sectional university-based “COSAMe” survey, which planned several measurement times, beginning on April 17, 2020. During this survey, and at each measurement interval, the French Ministry of Higher Education, Research, and Innovation asked all 82 universities in the French territory to contact their students (approximately 1,600,000 students) by email and to request that they participate by completing an online, self-administered questionnaire. Due to the heterogeneity of sanitary measures from one country to another, only students residing in France during the COVID-19 lockdown were included. The survey was anonymous, and no compensation was offered.

The first measurement time (T1) took place during the COVID-19 lockdown, between April 17 and May 4, 2020. The protocol and detailed results at T1 are available in a previous publication^6^. The second measurement time (T2) occurred 1 month after the quarantine was lifted between June 15 and July 15, 2020. At T2, nearly 30,000 deaths were attributed to COVID-19 in France.

The present study only included respondents to T2. Some of the participants had already responded to T1; the responses between T1 and T2 have been linked using a pseudonymization method.

This study was approved by a French research ethics committee, the Comité de Protection des Personnes Ile de France VIII, before its initiation.

### Collected data

We screened probable PTSD at T2 using the French version of the PCL-5, a 20-item scale that explores PTSD symptom severity over the past month^14^. The rating of the items ranges from 0 (“not at all”) to 4 (“extremely”), with a total score between 0 and 80, and a score higher than 32 leading to the potential diagnosis of PTSD^12,14^. The French version of the PCL-5 has good internal consistency, with a Cronbach’s alpha between 0.79 and 0.94^14^. We also assessed the prevalence of probable PTSD as defined by the DSM-5 criteria by retaining the presence of PTSD only among students reporting at least (a) one re-experiencing symptom, (b) one avoidance symptom, (c) two negative alterations in cognition or mood symptoms, and (d) two arousal symptoms (a symptom was considered present with a score above 1 on the PCL-5)^15^.

We collected several variables known to be linked to PTSD or that are likely to have worsened the quarantine experience to test their association with PTSD^1,9,16^. The variables available for all participants were:

- Socio-demographic characteristics: age, gender (male, female, other);
- Clinical information: a history of psychiatric follow-up, having experienced traumatic events not linked to COVID-19 after the beginning of the pandemic (i.e., likely to endanger the life or the physical or the psychological integrity of a person who is exposed to it)^17^;
- Social support characteristics: being a foreign student, living alone during the quarantine, the subjective quality of social relationships during the quarantine (rated out of 10 on an 11-point Likert-type scale), a feeling of being socially integrated before the quarantine (rated out of 10 on an 11-point Likert-type scale);
- Socio-economic factors: loss of income due to quarantine, subjective housing quality (rated out of 10 on an 11-point Likert-type scale);
- The subjective quality of the information received about the pandemic context and the associated sanitary measures (rated out of 10 on an 11-point Likert-type scale);
- Level of exposure to the COVID-19 pandemic: A COVID-19 exposure scale was constructed based on the methodology used by Tang et al.^9^.The scale includes seven items coded as yes or no: living in a worst-hit area (i.e., a department counting more than 50 deaths due to COVID-19 on March 29, 2020), having presented symptoms consistent with COVID-19 since the beginning of the pandemic, having been in contact with an infected person, having faced the death of a relative due to COVID-19, experiencing subjective fear for one’s health or the health of relatives (rated out of 10 on an 11-point Likert-type scale), being highly exposed to media messages related to the pandemic (in min per day). The last 3 items were rated yes when the score was greater than the 3rd quartile. The total score of the COVID-19 exposure scale was calculated by adding up the “yes” responses and was therefore rated from 0 to 7.

For students who also responded at T1, distress at T1 was also available, as assessed by the Impact of Events Scale-Revised (IES-R)^18^, a scale composed of 22 items rated by participants on a 5-point Likert scale to indicate the extent to which each of these items applies to their experiences during the preceding 7 days, from 0 (“not at all”) to 4 (“extremely”). The total score ranges from 0 to 88, and distress is considered normal for scores between 0 and 23, mild between 24 and 32, moderate between 33 and 36, and severe above 36^7^.

Finally, at T2, the participants had to indicate whether they considered the following pandemic-related events as traumatic (i.e., likely to endanger the life or the physical or psychological integrity of a person who is exposed to it^17^): news of a COVID-19 epidemic in China, news of a COVID-19 epidemic in France, the closure of shops, bars and meeting places, the closure of schools and universities, quarantine, having symptoms compatible with COVID-19, being infected with SARS-CoV2, being hospitalized for COVID-19, having a relative infected with SARS-CoV2, and having a relative hospitalized for COVID-19 and relatives who have passed away from COVID-19.

### Statistical analyses

Only participants who fully completed the questionnaires were analyzed.

The qualitative variables were summarized using percentages, and the quantitative variables were summarized using means and standard deviations or medians and interquartile ranges (IQRs), depending on whether they were normally distributed or not.

The prevalence rates of probable PTSD and their 95% confidence intervals were presented taking into account the two assessment methods. Cohen’s kappa coefficient was calculated to assess the concordance between the two methods. To identify factors associated with probable PTSD as assessed by a PCL-5 score above 32, multivariate logistic regression analysis (including all explanatory variables available for the whole sample) was performed. Associations between risk factors and outcomes are presented as odds ratios (ORs) and 95% confidence intervals (CI 95%).

Based on the subgroup of students who participated in both T1 and T2, assuming that the pandemic context and the associated lockdown may be a potentially traumatic event, we assessed the proportions of probable PTSD at T2 according to the level of distress at T1. The prevalence rates of the different psychological response trajectories as defined by Galatzer-Levy et al. (i.e., resilience, persistence, recovery, and delayed onset trajectories) were also calculated^8^. Trajectories were defined as follows: (a) resilience for students who had neither severe distress at T1 nor PTSD (as assessed by a PCL-5 score above 32) at T2, (b) persistence for students with both severe distress at T1 and PTSD at T2, (c) recovery for students with severe distress at T1, but who did not develop PTSD at T2 and (d) delayed onset for students who did not report severe distress at T1 but presented PTSD at T2.

Data analysis was carried out using R 3.6.1. The significance level was set at *α* = 0.05, and all tests were two-tailed.

## Results

### Sample characteristics

A total of 22,883 students completed the questionnaire at T2. Their characteristics are presented in Table 1.

**Table 1:** Characteristics of the sample and factors associated with probable PTSD in the global sample according to multivariate logistic regression analysis.

	Global sample *N* = 22,883		No probable PTSD *N* = 18,427		Probable PTSD *N* = 4456		Adjusted OR [CI 95%]		*p*
*Socio-demographic characteristics*									
Age, *m* (sd)^a^	20.9	(4.1)	20.8	(4.1)	21.2	(4.0)	0.98	[0.98–0.99]	0.003
Gender, *n* (%)									
Male	5906	(25.8)	4981	(27.0)	925	(20.8)	1	[ref]	
Female	16,640	(72.7)	13,232	(71.8)	3408	(76.5)	1.32	[1.21–1.45]	<0.001
Others	337	(1.5)	214	(1.2)	123	(2.8)	1.76	[1.35–2.31]	<0.001
*Clinical information*									
Psychiatric history, *n* (%)	2417	(10.6)	1467	(8.0)	950	(21.3)	2.26	[2.05–2.51]	<0.001
Exposure to another traumatic event, *n* (%)	3221	(14.1)	1745	(9.5)	1476	(33.1)	3.37	[3.08–3.67]	<0.001
*Social support*									
Living alone, *n* (%)	2646	(11.6)	1908	(10.4)	738	(16.6)	1.22	[1.09–1.37]	<0.001
Foreign student, *n* (%)	1365	(6.0)	898	(4.9)	467	(10.5)	1.70	[1.48–1.95]	<0.001
Quality of social ties, *n* (%)									
High (7–10)	9706	(42.4)	8474	(46.0)	1232	(27.6)	1	[ref]	
Medium (4–6)	8894	(38.9)	7126	(38.7)	1768	(39.7)	1.42	[1.30–1.55]	<0.001
Low (0–3)	4283	(18.7)	2827	(15.3)	1456	(32.7)	2.38	[2.15–2.62]	<0.001
Feeling integrated, *n* (%)									
High (7–10)	14,310	(62.5)	12,232	(66.4)	2078	(46.6)	1	[ref]	
Medium (4–6)	6872	(30.0)	5136	(27.9)	1736	(39.0)	1.56	[1.44–1.69]	<0.001
Low (0–3)	1701	(7.4)	1059	(5.7)	642	(14.4)	2.21	[1.95–2.51]	<0.001
*Socio-economic factors*									
Loss of income, *n* (%)	4184	(18.3)	3090	(16.8)	1094	(24.6)	1.20	[1.09–1.31]	<0.001
Housing quality, *n* (%)									
High (7–10)	19,229	(84.0)	16,052	(87.1)	3177	(71.3)	1	[ref]	
Medium (4–6)	2921	(12.8)	1961	(10.6)	960	(21.5)	1.60	[1.45–1.76]	<0.001
Low (0–3)	733	(3.2)	414	(2.2)	319	(7.2)	1.90	[1.59–2.26]	<0.001
Quality of information received, *n* (%)									
High (7–10)	8375	(36.6)	7082	(38.4)	1293	(29.0)	1	[ref]	
Medium (4–6)	10,586	(46.3)	8444	(45.8)	2142	(48.1)	1.26	[1.15–1.37]	<0.001
Low (0–3)	3922	(17.1)	2901	(15.7)	1021	(22.9)	1.50	[1.35–1.66]	<0.001
*Exposure score, n (%)*									
0	6386	(27.9)	5698	(30.9)	688	(15.4)	1	[ref]	
1	7159	(31.3)	6053	(32.8)	1106	(24.8)	1.38	[1.24–1.54]	<0.001
2	5057	(22.1)	3951	(21.4)	1106	(24.8)	2.02	[1.81–2.26]	<0.001
3	2757	(12.0)	1911	(10.4)	846	(19.0)	3.07	[2.71–3.47]	<0.001
4	1117	(4.9)	626	(3.4)	491	(11.0)	4.62	[3.95–5.41]	<0.001
5	330	(1.4)	158	(0.8)	172	(3.8)	6.87	[5.32–8.87]	<0.001
6	69	(0.3)	28	(0.1)	41	(0.9)	8.17	[4.79–14.06]	<0.001
7	8	(0.0)	2	(0.0)	6	(0.1)	10.82	[2.33–76.57]	0.005

^a^ *m* (sd) mean (standard deviation).

The majority of the respondents were women (72.7%). The average age was 21 (± 4). Regarding clinical information, 10.6% of the students reported a history of psychiatric follow-up, and 14.1% declared having been exposed to a traumatic event not related to the COVID-19 after the pandemic began.

As for social situations, 6.0% of the participants were foreign students, and 11.6% lived alone during the quarantine. The median score [IQR], given to the feeling of integration into society before the quarantine, was 7 [6–8] out of 10, and a median score of 6 [4–8] was attributed to the quality of the social bonds during the quarantine.

Regarding socio-economic factors, 18.3% of the students reported a loss of income related to the consequences of the pandemic. The median score given to the housing quality during lockdown was 9 [7–10]; a median score of 6 [4–7] was attributed to the quality of the information received during the lockdown.

Finally, concerning exposure to COVID-19 during the lockdown, 28.1% of the students resided in an affected department, 14.4% declared having been in contact with infected people, 5.8% reported knowing a person that had died from COVID-19, and 23.1% claimed to have had symptoms consistent with COVID-19. A median score of 7 [5–8] and 3 [1–5] was attributed to worries about a relative’s health and one’s own health, respectively. Students spent a median of 20 [5–45] min watching the news about the pandemic. In total, the median exposure score was 1 [0–2] out of 7.

### **PTSD** and associated factors

Among the participants, 4456 (19.5% CI 95% [19.0–20.0]) had probable PTSD as assessed by a PCL-5 score above 32, and 4449 (19.4% CI 95% [18.9–20.0]) reported all PTSD criteria as defined by the DSM-5. The observed agreement between the two assessment methods was 91.7% and the Kappa coefficient was 73.8% [CI 95%: 72.7–74.9].

In the multivariate analysis (Table 1), all factors were significantly associated with probable PSTD: (a) socio-demographic factors: age (OR [CI 95%] = 0.99 [0.98–0.99], *p* = 0.003), being a woman (1.32 [1.21–1.45], *p* < 0.001) or a non-binary person (1.76 [1.35–2.31], *p* < 0.001); (b) clinical factors: declaring a psychiatric history (2.26 [2.05–2.51], *p* < 0.001) or exposure to another traumatic event (3.37 [3.08–3.67], *p* < 0.001, respectively); (c) indicators of social support: having lived through quarantine alone (1.22 [1.09–1.37], *p* < 0.001), being a foreign student (1.70 [1.48–1.95], *p* < 0.001), a medium (1.42 [1.30–1.55], *p* < 0.001) or poor (2.38 [2.15–2.62], *p* < 0.001) quality of social ties compared to those declaring a good quality, and a medium (1.56 [1.44–1.69], *p* < 0.001) or low (2.21 [1.95–2.51], *p* < 0.001) feeling of integration compared to those reporting good integration; (d) indicators of precariousness: loss of income (1.20 [1.09–1.31], *p* < 0.001) and medium (1.60 [1.45–1.76], *p* < 0.001) or poor quality housing (1.90 [1.59–2.26], *p* < 0.001) compared to good quality housing; a medium or low-quality of information received compared to good quality (1.26 [1.15–1.37], *p* < 0.001 and 1.50 [1.35–1.66], *p* < 0.001, respectively); (e) level of exposure: the more the students were exposed to COVID-19, the more at-risk they were of probable PTSD (OR [CI 95%] from 1.38 [1.24–1.54], *p* < 0.001 for a score of 1 to 10.82 [2.33–76.57], *p* = 0.005 for a score of 7, vs a score of 0).

### Traumatic events

**Fig. 1:**
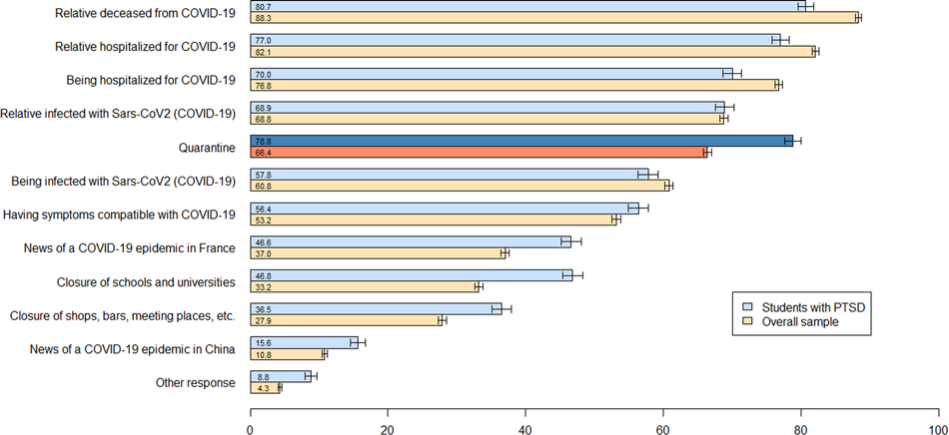
Proportions of students considering COVID-19-related events as potentially traumatic. A list of pandemic-related events was presented to the students. They were asked, for each event, to indicate if they considered it as traumatic (i.e., likely to endanger the life or the physical or psychological integrity of a person who is exposed to it). In yellow, the proportions of students answering “yes” among the whole sample. In blue, the proportions of students answering “yes” among those with a probable PTSD (as assessed by a PCL-5 score above 32). Quarantine, event of interest in our study, is highlighted.

For each event related to COVID-19, Fig. 1 shows the proportion of students who considered the event to be potentially traumatic. More than three-quarters of the students considered the direct consequences of a Sars-CoV2 infection to be traumatic, such as death (88.3%) or hospitalization, whether concerning a relative (82.1%) or oneself (76.8%). Most of the participants believed that the infection contracted by a relative (68.8%) or by oneself (60.8%) and that presenting symptoms compatible with COVID-19 could be traumatic (53.2%). Quarantine came in 5th position: two-thirds (66.4%) of students perceived it as potentially traumatic. Finally, a minority of respondents viewed the following events as potentially traumatic: news of the COVID-19 epidemic in France (37.0%), the closure of schools and universities (33.2%), the closure of bars, shops, and meeting places (27.9%), and news about the COVID-19 epidemic in China (10.8%).

Among the students presenting probable PTSD, the order was generally the same, except for the quarantine which came in the second position, just after the death of a relative (78.8% of them saw the quarantine as traumatic).

### Response trajectories

Among the 6947 students who responded to both T1 and T2, 1140 (16.4% CI 95% [15.5–17.3]) developed a probable PTSD at T2. Proportions of probable PTSD at T2 were 4.3% [3.7–5.0] for no symptoms of distress (as assessed by the IES-R score) at T1 (176 out of 4105 students), 17.1% [14.9–19.5] for mild symptoms (181 out of 1061), 25.0% [20.7–29.8] for moderate symptoms (93 out of 372), and 49.0% [46.3–51.6] for severe symptoms (690 out of 1409). Thus, the response trajectories were as follows: 1) resilience for 5088 students (73.2% [72.2–74.3]), 2) recovery for 719 students (10.3% [9.6–11.1]), 3) persistence for 690 students (9.9% CI 95% [9.2–10.7]); and 4) delayed onset for 450 students (6.5% [5.9–7.1]).

## Discussion

In the present study, 19.5% of French university students reported severe post-traumatic stress symptoms 1 month after the COVID-19 lockdown. Female status or non-binary gender, psychiatric follow-up history, exposure to a non-COVID-19-related traumatic event, having lived through quarantine alone, being a foreign student, poor quality of social ties, a low feeling of integration, loss of income, poor quality housing, low-quality of the information received, and a high level of exposure to COVID-19, were significantly associated with the severity of PTSD symptoms. Response trajectories to the context of the COVID-19 pandemic and the associated quarantine were resilience for 73.2% of the students, followed by recovery (10.3%), persistence (9.9%), and delayed onset trajectory (6.5%).

The rate of probable PTSD assessed in this study is much higher than the prevalence before quarantine, estimated at 0.7% in the French general population^19^. This suggests that the pandemic and lockdown period have a harmful impact on mental health, as highlighted by previous research on COVID-19^9,20,21^, as well as on quarantine measures during other epidemics^1^. It also strengthens concerns about the mental health of young adults, especially students, who were already identified as vulnerable before the pandemic^4^. The probable PTSD rate identified in our sample is higher than the PTSD rates in other populations: 7% in China’s hardest-hit areas according to Liu et al.^22^, 3.8% among front-line health care workers in China according to Yin et al.^23^, 1.2% among Canadian pregnant women according to Berthelot et al.^24^ and 7.0% in French patients with COVID-19^25^. All these studies used PCL-5 with a similar cut-off. Our results may thus reflect the greater vulnerability of students to the health crisis, compared to other population groups. Quarantine, in particular, may have been harder for young adults, occurring in a crucial period of their social development. The health crisis has profoundly shaken the health system, but also the education system. Social distancing and university closure during the COVID-19 pandemic may have reduced access to social support and mental health care and altered economic situations, in a population already vulnerable to mental health issues.

Risk factors associated with probable PTSD were the same as those associated with severe acute distress, uncovered during the first measurement time of the COSAMe survey. This is consistent with previous studies that have already pinpointed female gender, a history of mental health problems, poor social support, indicators of precariousness, and exposure to a traumatic event as risk factors for psychological disorders^1,7,9^.

We measured the concordance between the method identifying probable PTSD from a score greater than 32 at the PCL-5 and the one based on the presence of each DSM-5 criterion in response to the PCL-5 items. Both methods found equivalent prevalence rates with a high level of agreement. However, because the pandemic context or lockdown measures do not meet the requirements of diagnostic criterion A for PTSD, there is a debate as to whether the pandemic context and lockdown measures can lead to PTSD^3^. Students with severe distress (as assessed by the IES-R score) during confinement have a much higher probability of later probable PTSD than those with a lower score, suggesting the existence of confrontation with a traumatic event during confinement. Brunet et al. found that peri-traumatic distress predicts PTSD symptoms and diagnosis: in their study, elevated peri-traumatic distress was associated with a diagnosis of PTSD at 1, 6, and 12 months in more than 50% of the cases^26^. We found similar results, suggesting that IES-R could be a useful tool to identify at-risk students needing to be followed.

It has been proposed that high rates of PTSD among confined populations could be underpinned by the high incidence of traumatic events experienced in one’s home, but not because of the quarantine itself^27^. This study revealed that traumatic events unrelated to the pandemic were frequent (14.1%) and associated with an increased risk of probable PTSD. However, after adjusting for this variable, other factors related to the pandemic context remain associated with an increased risk of probable PTSD: both the pandemic itself (level of exposure to COVID-19) and the quarantine (isolation caused by confinement) appear to still play a role. Interestingly, quarantine was considered more potentially traumatic by the students than being infected with SARS-CoV2. This could be explained by the fact that, while young adults are less at-risk of the direct effects of COVID-19, they are particularly sensitive to the consequences of forced isolation related to quarantine measures^28^. Quarantine even came in second among students with probable PTSD, 78.8% of them considering the event as traumatic. This result further strengthens the role of quarantine measures in the occurrence of PTSD. The DSM-5 criterion A of PTSD assumes that only specific stressors (exposure to actual or threatened death, serious injury, or sexual violence) have “traumatic” characteristics. Exceptional contexts such as the COVID-19 pandemic and the unprecedented sanitary measures question the limits of this definition and stress the need to further explore the potential “traumatic” nature of complex events that are difficult to characterize with these restrictive criteria.

There are some limitations to our study. On the one hand, even though we used a validated diagnostic tool based on the DSM-5, unlike previous studies assessing the rates of PTSD in students, the PCL-5 only provides a provisional diagnosis of PTSD that should be confirmed by a clinician. Nevertheless, this scale indicated strong reliability and validity, and a cut-off of 31–33 was shown to be optimally effective in diagnosing PTSD^14^. We found a high level of agreement with the method considering PTSD diagnostic criteria according to the DSM-5. On the other hand, our results may have overestimated the PTSD prevalence due to self-selection bias. Indeed, we discovered a higher rate of probable PTSD in new respondents than in the subgroup already present at T1 (19.5% vs 16.4%); this suggests that students with mental health problems were more inclined to participate in mental health surveys at T2. However, it is also known that studies focusing on stigmatized behaviors or diseases are avoided by the people concerned^29,30^. In addition, the prevalence rate of PTSD measured in this study corresponds to that expected from the response trajectories to a potentially traumatic event (if we consider quarantine to be such an event)^8^. Finally, although the statistical model used is adapted to the study design, odds ratios should not be considered as risk ratios while interpreting the results. Indeed, as the prevalence rate of the outcome is quite high, the odds ratios may have overestimated the risk ratios.

In summary, we observed a high prevalence of probable PTSD among French university students one month after the end of the quarantine related to the COVID-19 pandemic. Importantly, these results suggest the pandemic context and lockdown measures could have a “traumatic” nature in this population, stimulating debate on the nosography of PTSD.
